# Computational Modeling of Microwave Tumor Ablation

**DOI:** 10.3390/bioengineering9110656

**Published:** 2022-11-05

**Authors:** Marija Radmilović-Radjenović, Nikola Bošković, Branislav Radjenović

**Affiliations:** Institute of Physics, University of Belgrade, Pregrevica 118, 11080 Belgrade, Serbia

**Keywords:** finite element method, microwave ablation, heat transport, tissue damage

## Abstract

Microwave ablation is recognized as a minimally invasive, fast-recovery treatment for destroying cancer cells using the heat generated by microwave energy. Despite the unquestionable benefits of microwave ablation, the interaction of the microwave applicator with the tissue may result in localized heating and damage to the surrounding tissue. The majority of the tissue damage can be removed by clarifying the conditions for their development. In addition to experimental methods, computer modeling has proven to be an effective tool for optimizing the performance of microwave ablation. Furthermore, because the thermal spread in biological tissue is difficult to measure, developing a predictive model from procedural planning to execution may have a substantial influence on patient care. The comprehension of heat transport in biological tissues plays a significant role in gaining insight into the mechanisms underlying microwave ablation. Numerical methods that enable ablation size control are required to guarantee tumor destruction and minimize damage to healthy tissues. Various values of input power and ablation time correspond to different tumor shapes ensuring the preservation of healthy tissues. The optimal conditions can be estimated by performing full three-dimensional simulations. This topical review recapitulates numerous computational studies on microwave tumor ablation. Novel areas emerging in treatment planning that exploit the advantages of numerical methods are also discussed. As an illustration, the results of the three-dimensional simulations of real liver tumors in the 3D-IRCADb-01 database are presented and analyzed. The simulation results confirm that numerical methods are very useful tools for modeling microwave tumor ablation with minimal invasiveness and collateral damage.

## 1. Introduction

Microwave ablation (MWA) is the most widely used ablation method for a broad spectrum of cellular pathologies and malignancies [1,2,3,4,5]. MWA is a thermal modality based on tissue destruction by localized heat, which increases the temperature to exceed the physiological threshold to kill cancer cells. Compared with other thermal technologies, MWA offers unique advantages such as an improved convection profile, higher intratumoral temperatures, larger ablation zones especially when using multiple antennas simultaneously, and reduced treatment times [6,7,8,9,10,11,12]. In particular, MWA at 2.45 GHz has become the highly recommended treatment for primary and secondary liver malignancies with both curative and palliative intent. According to the literature, MWA treatment completely eliminates small liver tumors, with a success rate greater than 85% [13]. During MWA, ablation can be accomplished by either implicitly controlling the power or temperature, resulting in a modulating heat procedure [14]. 

**Figure 1:**
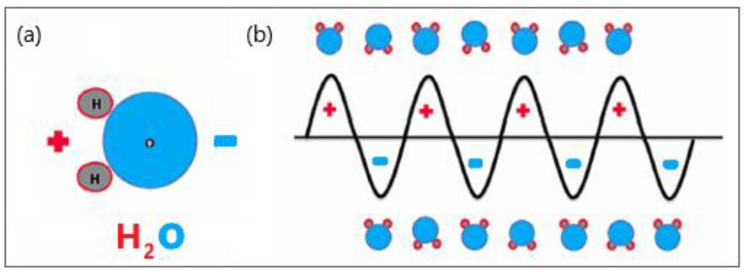
Schematic illustration of (**a**) polar water molecules and (**b**) interaction between an oscillating electromagnetic microwave waveform and water molecules.

Microwave (MW) antennas that operate at microwave frequencies (915–2450 MHz) and radiate microwave energy are strategically placed in biological tissues [15,16]. The MW field causes the rotation of polar molecules, therefore, part of the electromagnetic (EM) energy is absorbed and converted into heat. Water molecules are polar, and therefore, the electric charges on these molecules are asymmetric. Hydrogen atoms are positively charged, whereas oxygen atoms have a negative charge, represented by red and blue, respectively (see Figure 1a). The electric charge of electromagnetic radiation flips between the positive and negative values. The MW field oscillates rapidly, causing water molecules to spin and change orientation 2–5 billion times per second depending on the frequency of the MW energy, as shown in Figure 1b [17]. The vigorous molecular movement of water molecules significantly raises the temperature of the water, heating the target tissue and causing cancer cell death via coagulation necrosis [18].

Understanding the dielectric properties of tissues is particularly important for the application of MWA [19,20,21]. Electrical conductivity *σ* is the current density per unit electric field, with units of kg^−1^n^−3^s^3^A^2^. Current density is related to ion mobility, which is velocity per unit electric field. The physical characteristic that determines MW penetration in tissues is related to permittivity *ε* and it is given by the relation $\varepsilon =\varepsilon^{\prime }-j\varepsilon^{\prime \prime }=\varepsilon_{r}-j\sigma /\omega \varepsilon_{0},$ where *ω* is the angular frequency, *ε_r_* is the static relative permittivity, and *ε*_0_ is the vacuum permittivity [22]. It has been reported that the dielectric properties of tumoral tissues are 10–20% greater than those of healthy tissues [23,24].

Another factor that strongly affects the efficiency of MWA is the antenna design, which is responsible for the formation of ablation zones that extend radially from the antenna. Over the past few decades, various antenna designs have been developed to minimize thermal damage to healthy tissue [25,26,27,28]. Early-generation MWA antennas created elongated ablation zones with the so-called ‘comet tail’, which invades the healthy surrounding tissue [29]. Recent antenna designs have produced shorter ablation zones centered on the antenna tip, which is useful for treating small tumors. [30,31]. Some commercial MWA systems based on “thermosphere technology” can produce spherical ablation patterns by adding small saline irrigation channels to the antennas [32,33]. Novel compact multislot coaxial antennas ensure the formation of spherical ablation zones that are suitable for the interstitial MWA process [34,35].

In addition to experimental methods, computer modeling is a powerful tool for improving the performance of MWA devices and procedures. Moreover, numerical models of the antenna-tissue system are essential for investigating thermal spread through the tissue [36,37,38,39]. Several numerical studies have predicted the temperature profile and accompanying tissue damage caused by the MWA applicator [40,41]. The Arrhenius model is widely employed to estimate the degree of tissue destruction [42,43]. Owing to the complexity of the models and computational resource consumption, the majority of actual numerical studies are based on two-dimensional (2D) axisymmetric models to reproduce realistic tumor shapes, which are usually non-axisymmetric [44,45,46,47,48]. Several 3D simulations have recently been performed to determine the fraction of damage during MWA of tumoral tissues [49,50,51,52,53]. The higher the level of predictability achieved in simulations, the easier it is to plan a treatment procedure that is safer, more effective, and less time-consuming.

In this topical review article, we focus primarily on numerical methods useful in modeling the MWA treatment of tumors. Although computational modeling has experienced unprecedented growth in recent years owing to the availability of high-performance low-cost computers, attention needs to be paid to the actual capabilities and limitations of current simulation techniques. This article is organized as follows. The introduction describes the fundamentals of the MWA procedure. The advances and limitations of various mathematical models of tissue and heat transport through tissues are discussed in Section 2. Special attention will be paid to progress in the development of heat-transfer equations in biological tissues. Section 3 describes the modeling of the electromagnetic field generated by an antenna immersed in a tissue. Certain simulation results regarding the modeling of the MWA of hepatocellular carcinoma will be highlighted in Section 4 helping us to understand the application of numerical methods in biomedicine in more detail. Section 5 reflects the author’s point of view regarding computer modeling of microwave tumor ablation. We conclude this article in Section 6, indicating perspectives on new studies and methodology directions that have begun to appear as numerical models have been developed more completely. 

## 2. Mathematical Models of Tissue and Heat Transport

In recent years, numerical models of ablation procedures have undergone continuous refinement and have been adopted to simulate the effects of current flow through biological tissue [54,55,56,57]. Furthermore, thermal spread in biological tissues is difficult to measure; hence, a predictive model of the MWA procedure can significantly improve the efficiency of this thermal modality. Mathematical models of MWA procedures consist of three fundamental components. The first is the modeling of tissue and heat transport in tissues. The second is related to the modeling of the microwave EM field generated by ablation probes (antennas). The third component is associated with modeling the effect of heating on tumor cells.

Kenyon [58] applied the mixture theory to biological tissues, while experimental investigations of biological tissues within a theoretical framework began in earnest in a series of papers [59,60,61,62,63]. One of the main challenges in using mixture theory as a modeling framework for biological tissues is the apparent complexity. Cowin [64] concluded that most studies related to the mixture theory have an unusually large number of equations. As the structure of biological tissues is porous and consists of different cells and a microvascular bed, the theory of porous media for heat transfer in tissues is more appropriate than that of a homogenous model [65,66]. Most previous models of heat transport have focused on single-layer porous media biomaterials [67,68,69]. According to porous media theory, the entire biological medium can be divided into distinct tissue and blood phases. The governing equations for bioheat transfer and blood flow were averaged over the control volume. 

In studies that used porous media models for heat transfer in biomaterials, various parameters, such as tumor diameter, tumor porosity, and input MW power were not considered. However, in practice, these parameters may intensify the absorption process in the target tissue. Hence, to represent the actual process of MW ablation, it is crucial to take into account complete modeling based on the porous media theory in porous tissue.

### 2.1. Tissue Model

**Figure 2:**
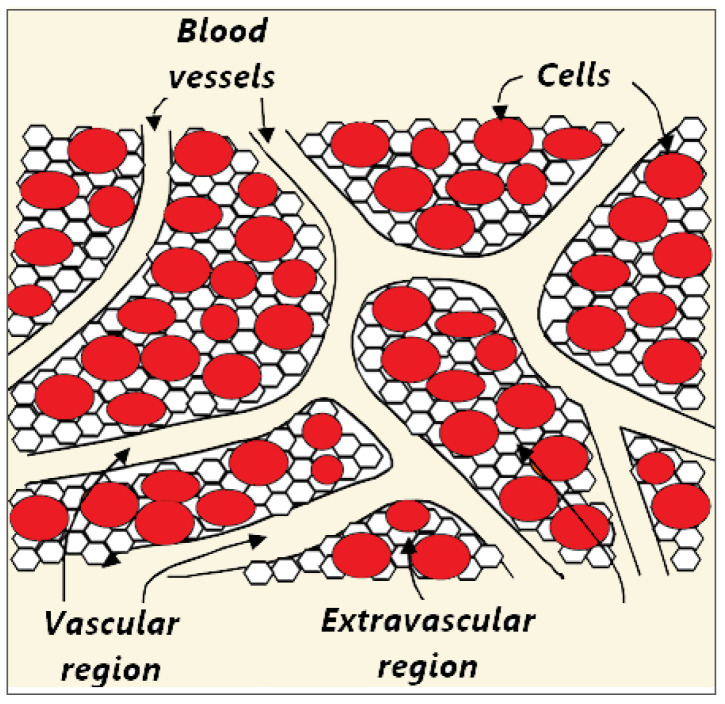
Schematic illustration of three compartments (blood vessels, cells, and interstitium) of the porous biological tissue. The interstitial space is split into the extracellular matrix and interstitial fluid.

Biological tissue is a complex heterogeneous system consisting of dispersed cells segregated by voids. Blood flows through a network of vessels, known as the circulatory system, which is composed of arteries, veins, and capillaries. The primary function of capillaries, the smallest and most numerous blood vessels, is the exchange of materials between blood and tissue cells. Figure 2 shows a schematic plot of the tissue consisting of blood vessels, cells, and interstitium, which can be further divided into the extracellular matrix and interstitial fluid [70,71]. However, for simplicity, biological tissue can be divided into two distinct regions: The vascular and extravascular regions (cells and interstitium). The extravascular region is considered to be a solid matrix, albeit with extravascular fluid. Thus, the tissue can be treated as a fluid-saturated porous medium through which the blood infiltrates.

The modeling of heat transport in biological tissues must include tissue heat exchange, blood-tissue convection, blood perfusion, and metabolic heat production [72]. Heat transport in tissues can be considered from different viewpoints: Molecular, microscopic, or macroscopic [73]. Moreover, heating may lead to vaporization (phase changes) and ionization of the water content and, in some cases, vapor expansion and tissue fragmentation. Therefore, microscopic modeling is practically impossible, and macroscopic modeling must be used for heat transport in tissues. 

The macroscale is a phenomenological scale that is far larger than the microscale of cells and voids, but substantially smaller than the system length scale. Macroscale considerations are important because of the possible complications caused by a large number of particles at the molecular scale or the complex microscale structure of biological tissues [74,75]. Good separation of the length scales is pivotal for establishing a macroscale representation identical to the microscale behavior [76,77]. There are two approaches to the development of macroscopic thermal models for blood-perfused tissues. The first is scaling down based on the mixture theory of continuum mechanics. The second one is recognized as scaling up from the microscale based on the porous media theory [78].

#### 2.1.1. Top-Down Approach

The top-down approach does not involve microscale system representation or microscale quantities. The phase properties were determined at a macroscale level. The shortcomings of this approach are the failure to link microscale and macroscale properties and the extension of multiphase systems with different features of interfaces and common curves. Full balance equations are established in terms of macroscale properties, with additional terms related to the blood–tissue interaction. The energy conservation equation is expressed as [78,79]:

(1) $$\begin{array}{l} \frac{\partial {\left(\rho c\varepsilon T\right)}_{mac}^{t}}{\partial t}=-\nabla \cdot {\left(\varepsilon q\right)}_{mac}^{t}+{\left(\rho \varepsilon q_{m}\right)}_{mac}^{t}\\ +{\left(\rho \varepsilon q_{c}\right)}_{mac}^{t}+{\left(\rho \varepsilon q_{p}\right)}_{mac}^{t}+{\left(\rho \varepsilon q_{e}\right)}_{mac}^{t}, \end{array}$$

where *t* is the time, *T* is the temperature, ρ is the density, *ε* is the volume fraction, and *c* is the specific heat. Subscript “*mac*” denotes the macroscale properties, while the superscript *t* designates the tissue properties. The volumetric rates of heat due to metabolic heating, blood interfacial convective heat transfer, and blood perfusion are labeled by *q_m_*, *q_c_*, and *q_p_*, respectively. *q_e_* is the volumetric rate related to the external heat supply. For the heat flux density vector **q**, three constitutive relations can be used: The Fourier law, Cattaneo–Vernotte (CV) relation, and dual-phase-lagging (DPL) relation (more details can be found in Ref. [78]). 

Combining Fourier’s law (q(r,t)=−k∇T(r,t), where ***r*** denotes the material point and *k* is the thermal conductivity of the material) and Equation (1) leads to a group of thermal models for biological tissues (bioheat equations) [78]:

(2) $$\begin{array}{l} \frac{\partial {\left(\rho c\varepsilon T\right)}_{mac}^{t}}{\partial t}=\nabla \cdot {\left(\varepsilon k\nabla T\right)}_{mac}^{t}+{\left(\rho \varepsilon q_{m}\right)}_{mac}^{t}\\ +{\left(\rho \varepsilon q_{c}\right)}_{mac}^{t}+{\left(\rho \varepsilon q_{p}\right)}_{mac}^{t}+{\left(\rho \varepsilon q_{e}\right)}_{mac}^{t}. \end{array}$$

Using the CV constitutive relation $\left(q\left(r,t\right)+\tau_{q}\partial q\left(r,t\right)/\partial t=-k\nabla T\left(r,t\right)\right)$ and Equation (1) yields the following expression [78]:

(3) $$\begin{array}{l} \frac{\partial {\left(\rho c\varepsilon T\right)}_{mac}^{t}}{\partial t}+\tau_{q}\frac{\partial^{2}{\left(\rho c\varepsilon T\right)}_{mac}^{t}}{\partial t^{2}}=\nabla \cdot {\left(\varepsilon k\nabla T\right)}_{mac}^{t}\\ +\left(1+\tau_{q}\frac{\partial }{\partial t}\right){\left(\rho \varepsilon q_{m}+\rho \varepsilon q_{c}+\rho \varepsilon q_{p}+\rho \varepsilon q_{e}\right)}_{mac}^{t}. \end{array}$$

Finally, by combining the DPL constitutive relation $\left(q\left(r,t+\tau_{q}\right)=-k\nabla T\left(r,t+\tau_{T}\right)\right)$ with Equation (1), a hyperbolic bioheat equation is obtained [78]:

(4) $$\begin{array}{l} \frac{\partial {\left(\rho c\varepsilon T\right)}_{mac}^{t}}{\partial t}+\tau_{q}\frac{\partial^{2}{\left(\rho c\varepsilon T\right)}_{mac}^{t}}{\partial t^{2}}=\nabla \cdot \left[{\left(\varepsilon k\right)}_{mac}^{t}\nabla T_{mac}^{t}\right]+\tau_{T}\frac{\partial }{\partial t}\left\{\nabla \cdot \left[{\left(\varepsilon k\right)}_{mac}^{t}\nabla T_{mac}^{t}\right]\right\}\\ +\left(1+\tau_{q}\frac{\partial }{\partial t}\right){\left(\rho \varepsilon q_{m}+\rho \varepsilon q_{c}+\rho \varepsilon q_{p}+\rho \varepsilon q_{e}\right)}_{mac}^{t}. \end{array}$$

Equation (4) predicts that the temperatures and thermal tissue damage differ significantly from those obtained by the Pennes model [80,81,82]. 

#### 2.1.2. Bottom-Up Approach

**Figure 3:**
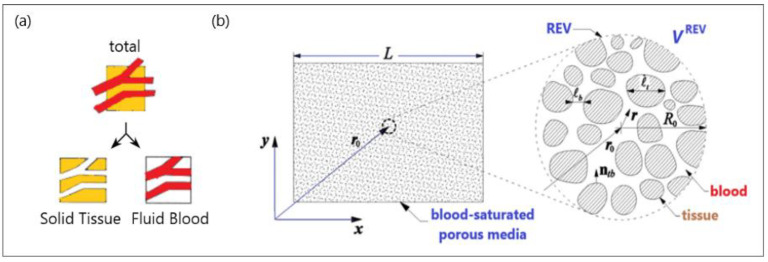
A schematic diagram of (**a**) a biological medium split into solid and blood phases and (**b**) a blood-saturated porous matrix encompassing cells and interstices and representative elementary volume (REV).

The basic idea behind the porous media theory is that the entire biological medium can be divided into two distinct phases corresponding to tissue and blood (see Figure 3a). The tissue phase is solid, involving cells and interstitial spaces. The blood phase is the fluid part considered as the blood, which flows through the solid phase [83]. The blood volume fraction in the whole biological medium is represented by porosity, which is very important for tissues with high vascularization (e.g., the kidney, liver, and tumors). 

To establish the macroscale equations governing blood flow and bioheat transfer, biological tissue is simplified to a blood-saturated porous matrix comprising cells and interstices, as displayed in Figure 3b [84]. The resulting microscale field equations were then averaged over a representative elementary volume *V*^REV^ [78,85]:

(5) $$\frac{1}{V^{\mathrm{REV}}}\int_{V^{b}}\nabla \cdot v_{mic}^{b}dV=0,$$

(6) $$\begin{array}{l} \frac{1}{V^{\mathrm{REV}}}\int_{V^{b}}{\left(\rho c\right)}_{mic}^{b}\frac{\partial T_{mic}^{b}}{\partial t}dV+\frac{1}{V^{\mathrm{REV}}}\int_{V^{b}}{\left(\rho c\right)}_{mic}^{b}v_{mic}^{b}\cdot \nabla T_{mic}^{b}dV\\ =\frac{1}{V^{\mathrm{REV}}}\int_{V^{b}}\nabla \cdot \left(k_{mic}^{b}\nabla T_{mic}^{b}\right)dV. \end{array}$$

(7) $$\begin{array}{l} \frac{1}{V^{\mathrm{REV}}}\int_{V^{t}}{\left(\rho c\right)}_{mic}^{t}\frac{\partial T_{mic}^{t}}{\partial t}dV=\frac{1}{V^{\mathrm{REV}}}\int_{V^{t}}\nabla \cdot \left(k_{mic}^{t}\nabla T_{mic}^{t}\right)\\ +\frac{1}{V^{\mathrm{REV}}}\int_{V^{t}}{\left(q_{m}\right)}_{mic}^{t}dV. \end{array}$$

where *V_b_* and *V_t_* are the blood (denoted by superscript *b*) and tissue (denoted by superscript *t*), respectively, in the REV. The subscript “mic” refers to microscale properties, **v** is the velocity, and *T* is the temperature. *ρ*, *μ*, *c*, and *k* are the density, viscosity, specific heat, and thermal conductivity, respectively. The volumetric rate of heat generated by the metabolic reaction is *q_m_*. 

### 2.2. Heat Transport in Tissues

MWA uses heat from microwave energy to kill cancer cells. The energy from the MW frequency waves emitted by the microwave coaxial antenna (MCA) creates heat in cancerous tissue without damaging the surrounding healthy tissue. As previously mentioned, the applied MW energy causes water molecules to vibrate and rotate, resulting in a temperature sufficiently high to cause cell death. An exact representation of heat transport through biological tissues plays a key role not only in the fundamental understanding of the process but also in many medical therapeutic options.

**Figure 4:**
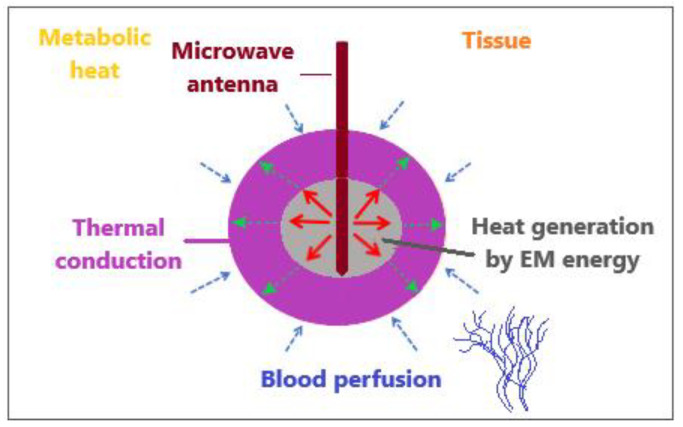
An illustrative overview of heat transfer mechanisms when an MW antenna is inserted into tissue to generate heat by the deposition of electromagnetic energy.

The bioheat transfer mechanism is illustrated in Figure 4 [86]. MW antenna immersed in tissue generates heat through the deposition of electromagnetic (EM) energy. Thermal diffusion of heat within the tissue leads to thermal conduction. Tissue fluid convection is unimportant for heat transfer inside the tissue because tissue fluid is either intracellular and cannot pass through the cell membrane or is extracellular with movement constrained by neighboring cells and the tissue matrix. Metabolic heat and blood perfusion are two mechanisms specific to biological tissues. Metabolic heat is generated by the metabolic activities of cells [87]. Blood perfusion causes the cooling of any tissue above the physiological temperature and, in many cases, represents a dominant mechanism affecting the tissue temperature during electromagnetic-energy-based thermal therapies [88]. 

Most studies devoted to MWA have dealt with Pennes’ bioheat equation for modeling the heat transport of biological tissues because of its simplicity despite certain shortcomings. [83]. This model was developed under the assumption of a uniform perfusion rate without considering the blood flow direction and artery–vein countercurrent arrangement. In Pennes’ bioheat model, all heat exchange between the tissue and vasculature occurs in the capillaries, and the temperature of the vasculature within the capillaries is equivalent to the core temperature of the human body. The assumptions also include that the venous blood is in thermal equilibrium with the tissue and that the arterial blood remains at a constant temperature of 37 °C. To overcome these simplifications and limitations, Pennes’ bioheat model has been extended, modified, or coupled with other models [83]. Some researchers have attempted to overcome the simplifications and limitations of this model in order to obtain more accurate results. Penne’s theory has been extended in numerous papers [89,90,91,92]. An extensive review of the different bioheat models can be found in Andreozzi et al. [14].

#### 2.2.1. Pennes Model

Heat transfer is often governed by the Pennes bio-heat equation [80,83]:

(8) $${\left(\rho c\right)}_{t}\frac{\partial T_{t}}{\partial t}=\nabla \times \left(k_{t}\nabla T_{t}\right)+\beta \rho_{b}\omega_{b}c_{b}\left(T_{b}-T_{t}\right)+Q_{\mathrm{ext}}+Q_{m},$$

where *t* is time. *ρ_t_*, *c_t_*, and *T_t_* are the density, heat capacity, and temperature of the tissue, respectively, and *ρ*_b_, *T*_b,_ *c*_b,_ and *w*_b_ are the density, temperature, heat capacity, and blood perfusion rate, respectively. The thermal damage affects values *β* between 0 and 1. The external heat source *Q*_ext_ refers to coupling with the electromagnetic field, whereas *Q*_m_ is related to metabolic heating [83].

Vaporization is embedded into Pennes’ equation via the enthalpy method, and the term (*ρ_t_c_t_*) in Equation (8) is given by the relation [83]

(9) $$\begin{array}{l} {\left(\rho c\right)}_{t}=\left\{ \begin{array}{l} {\left(\rho_{l}c_{l}\right)}_{t}\;\;0\;{}^{\circ}C<T_{t}\leq 99\;{}^{\circ}C\\ \frac{h_{fg}C_{w,t}}{\Delta T_{b,t}}\;\;99\;{}^{\circ}C<T_{t}\leq 100\;{}^{\circ}C\\ \rho_{g}c_{g}\;\;T_{t}>100\;{}^{\circ}C \end{array}\right. \end{array}$$

where *ρ_l_* and *c_l_* are the density and specific heat of tissue at a temperature below 100 °C (liquid phase). At temperatures above 100 °C (gas phase), the density and specific heat of the tissue are denoted by *ρ_g_* and *c_g_*, respectively. The product of the latent heat of vaporization and the water density at 100 °C is *h_fg_*, whereas *C_w_*_,*t*_ is the water content inside the liver tissue. 

#### 2.2.2. Modified Local Thermal Non-Equilibrium (LTNE) Model

The basic concept of the LTNE model is that the entire biological medium is divided into the tissue and blood phases. Thus, there are two energy equations for the LTNE model corresponding to the tissue temperature (*T_t_*) and blood temperature (*T_b_*) [83,93]:

(10) $$\begin{array}{l} \left(1-\varepsilon \right){\left(\rho c\right)}_{t}\frac{\partial \langle T_{t}\rangle }{\partial t}=\left(1-\varepsilon \right)k_{t}\nabla^{2}\langle T_{t}\rangle -h_{c}a\left(\langle T_{t}\rangle -\langle T_{b}\rangle \right)\\ +\beta \rho_{b}\omega_{b}c_{b}\left(\langle T_{b}\rangle -\langle T_{t}\rangle \right)+\left(1-\varepsilon \right)Q_{\mathrm{ext}}, \end{array}$$

(11) $$\begin{array}{l} \varepsilon {\left(\rho c\right)}_{b}\left(\frac{\partial \langle T_{b}\rangle }{\partial t}+\beta \langle u\rangle \cdot \nabla \langle T_{b}\rangle \right)=\varepsilon k_{b}\nabla^{2}\langle T_{b}\rangle +h_{c}a\left(\langle T_{t}\rangle -\langle T_{b}\rangle \right)\\ +\beta \rho_{b}\omega_{b}c_{b}\left(\langle T_{t}\rangle -\langle T_{b}\rangle \right)+\varepsilon Q_{\mathrm{ext}}, \end{array}$$

where *ɛ* is the ratio of the blood volume to the total volume, **u** is the vector of the blood velocity, and *β* is a coefficient whose value depends on the thermal damage function. *h_c_* is the interfacial heat transfer coefficient and is the volumetric heat transfer area between the tissue and blood phases. Vaporization is included for the tissue phase, whereas for the blood phase, the following relation can be used [83]:

(12) $$\begin{array}{l} {\left(\rho c\right)}_{b}=\left\{ \begin{array}{l} {\left(\rho_{l}c_{l}\right)}_{b}\;0\;{}^{\circ}C<T_{b}\leq 99\;{}^{\circ}C\\ \frac{h_{fg}C_{w,b}}{\left(100\;{}^{\circ}C-99\;{}^{\circ}C\right)}\;99\;{}^{\circ}C<T_{b}\leq 100\;{}^{\circ}C\\ \rho_{g}c_{g}\;T_{b}>100\;{}^{\circ}C \end{array}\right. \end{array}$$

where *C_w,b_* represents the water content in blood.

#### 2.2.3. Modified Local Thermal Equilibrium (LTE) Model

The LTNE model also describes blood and tissue phases at two distinct temperatures (i.e., *T_t_* ≠ *T_b_*). However, under the local thermal equilibrium hypothesis, the tissue and blood are at the same temperature (*T_t_ = T_b_ = T*); thus, Equations (10) and (11) can be combined into a single equation [83]:

(13) $$\begin{array}{l} \left[\left(1-\varepsilon \right){\left(\rho c\right)}_{t}+\varepsilon {\left(\rho c\right)}_{b}\right]\frac{\partial T}{\partial t}+\varepsilon {\left(\rho c\right)}_{b}\beta \cdot u\cdot \nabla T\\ =\left[\left(1-\varepsilon \right)k_{t}+\varepsilon k_{b}\right]\left(1-\varepsilon \right)\nabla^{2}T+Q_{\mathrm{ext}}. \end{array}$$

Although local thermal equilibrium is a good approximation for temperature distributions in small vessels, it is not valid for larger vessels. 

**Figure 5:**
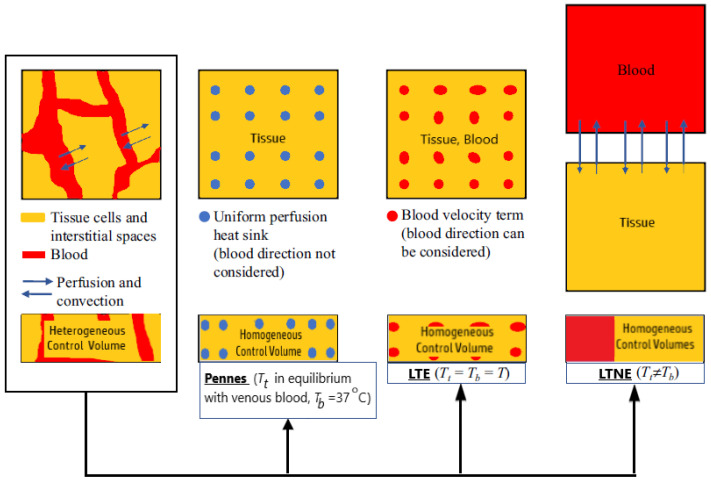
A comparison between Pennes’ model and two porous-media-based models (the Local Thermal Non-Equilibrium and the Local Thermal Equilibrium equations).

Dissimilarities between Pennes’ model and the two porous media-based models (the LTNE and the LTE equations) can be observed in Figure 5 [83]. In the LTE and LTNE models, the Pennes’ equation perfusion term is divided into a modified perfusion term and a convective term. Both models were modified to account for two-phase water vaporization (tissue and blood). 

## 3. Modeling of the Electromagnetic Field Generated by the Antenna

**Figure 6:**
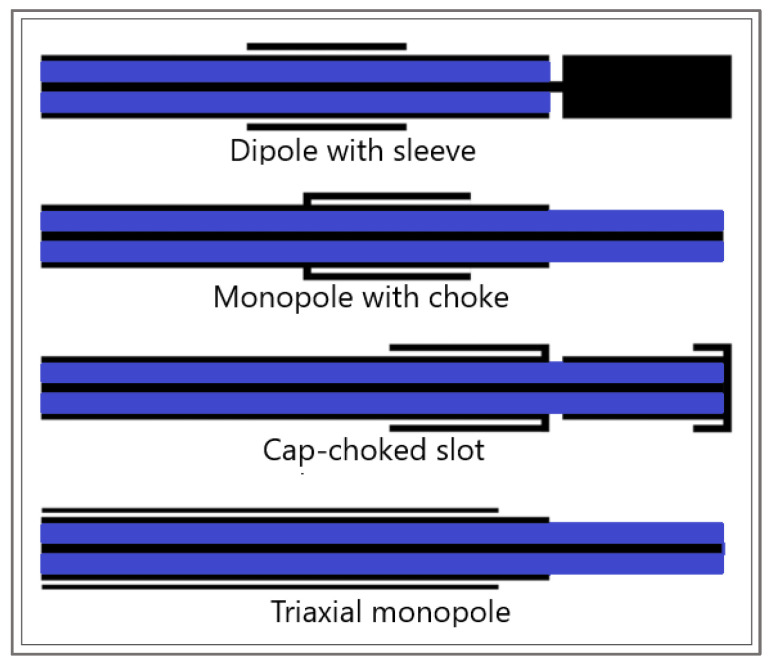
Sketches of antenna designs with sleeve, choke, cap-choked, and monopole in triaxial configuration. The metal and the dielectric are shown in black and blue, respectively.

**Figure 7:**
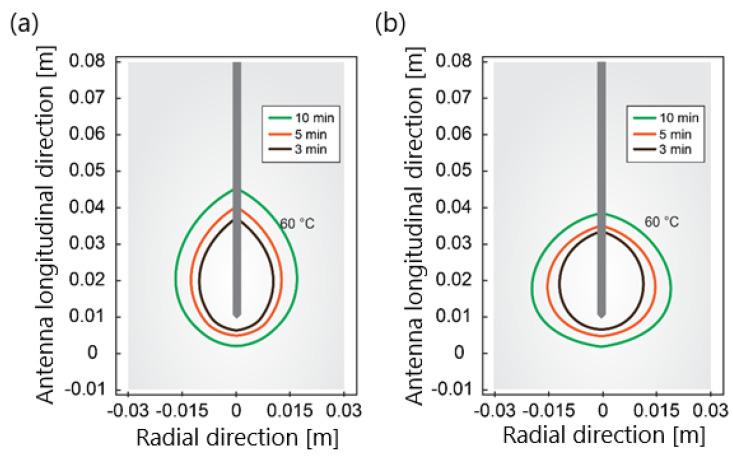
The isotherm corresponds to 60 °C for (**a**) a single−slot antenna and (**b**) 10−slot antenna with impedance matching after ablation times of 180 s, 300 s, and 600 s for an input power of 30 W.

Another component that is indispensable in any mathematical model of MWA is the modeling of the microwave EM field generated by ablation probes (antennas). The efficiency of the MWA procedure strongly depends on the structure of the MW antenna. The MW antenna delivers energy to the tumor and surrounding area, resulting in an increase in temperature to a lethal level and cell death in the ablation zone. To enhance the performance of the MWA procedure, various antenna designs have been developed including choke [94], cap-choke [95], floating sleeve [96,97], and water-cooled [98] antennas (Figure 6). The factors that may lead to a temperature increase along the antenna shaft and, thus, to the formation of elongated heating patterns (see Figure 7a) are the impedance mismatch between the antenna and tissues and the leakage current along the outer conductor of the antenna. Recently, small microwave antennas capable of creating larger spherical ablation volumes in the liver have been developed [95,99,100]. With its simple construction, compact size, and low cost, the coaxial antenna with several slots became a promising antenna design due to its simple construction, compact size, and low cost [99]. In their theoretical work, Wang et al. proposed a multi-slot coaxial antenna with periodic slots [99]. The desired shape of ablation zones can be attained by regulating the distance between adjacent slots and the number of slots. Compared with other antenna designs (see [101] and references therein), a multi-slot coaxial antenna has a better ability to produce an optimal shape of the ablation zone pattern (see Figure 7b) [101]. Recent simulation results have confirmed that a compact 10-slot antenna consisting of several periodic elements equivalent to a linear uniform antenna array creates near-spherical ablation zones [50,53]. Finely tuned impedance matching minimizes damage to the surrounding healthy tissues. In one of the most sophisticated antenna designs, the required ablation zones are achieved using three different mechanisms: Thermal, field, and wavelength controls [32,33].

**Figure 8:**
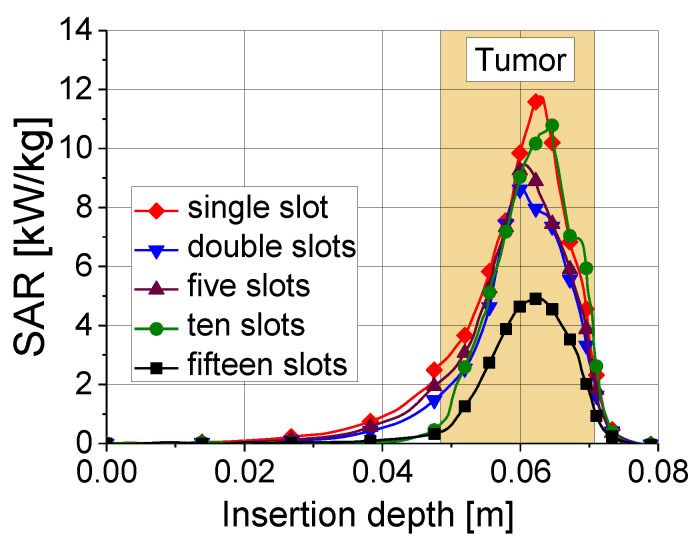
Specific absorption rate versus insertion depth for five antennas with different numbers of slots. The results were obtained for an input power of 45 W and ablation time of 600 s.

Tissue heating is directly related to the ratio of absorbed heat power to tissue density, that is, the so-called specific absorption rate (SAR). In the tumor region, the values of the SAR are maximal. As shown in Figure 8, a 10-slot antenna has the highest SAR value among antennas with various numbers of slots. 

Owing to its mechanical and geometrical complexity, antenna modeling is strongly affected by electromagnetic material and tissue properties. The antenna probe (or applicator) immersed in the tissue generates a microwave field governed by equations [50,102]:

(14) $$\nabla^{2}E-\mu_{r}k_{0}^{2}\left(\varepsilon_{r}-\frac{j\sigma }{\omega \varepsilon_{0}}\right)E=0,$$

where *ω* is the angular frequency, ***E*** is the electric field vector, *k*_0_ = *ω*/*c*_0_ is the vacuum propagation constant, and *ε*_0_ is the vacuum dielectric constant. *σ,* ε*_r_*, and *μ_r_* denote tissue electrical conductivity, relative permittivity, and tissue permeability, respectively.

**Figure 9:**
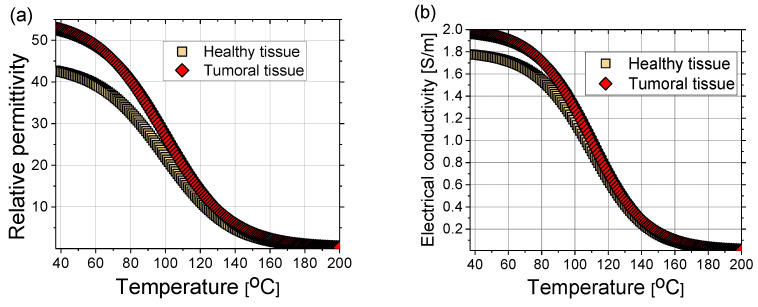
(**a**) Relative permittivity and (**b**) electrical conductivity of healthy (squares) and tumoral (diamonds) liver tissue as a function of time according to Equations (15) and (16) with coefficients from Ref. [102].

During MWA, an increase in the temperature may result in structural modifications of the treated tissue, leading to changes in the dielectric and thermal properties, thus affecting the electromagnetic power distribution [103]. The conductivity and permittivity depend on the temperature. The temperature dependence of tissue dielectric properties arises primarily because of the significant water concentration in organ tissue [104]. During MWA, tissue heating causes the evaporation of water molecules, which irreversibly changes the protein structures, causing changes in the conductivity and permittivity of the tissue [50,102].

(15) $$\varepsilon_{r}\left(T\right)=s_{1}\left[1-\frac{1}{1+\exp\left(s_{2}-s_{3}T\right)}\right],$$

(16) $$\sigma \left(T\right)=r_{1}\left[1-\frac{1}{1+\exp\left(r_{2}-r_{3}T\right)}\right],$$

where the coefficients can be found elsewhere (e.g., in [102]). The sigmoidal temperature-dependent model of (a) relative permittivity and (b) electric conductivity of healthy and tumoral liver tissues are plotted in Figure 9. Tumors have an approximately 24% higher relative permittivity and 11% higher conductivity than healthy tissue [105]. During MWA, as the temperature increases, water evaporation leads to a decrease in dielectric properties [102,106]. The time dependence of the water content *W(T)* is expressed as [40]:

(17) $$\begin{array}{l} W(T)=\left\{ \begin{array}{l} 0.778\cdot \left(1-e^{\frac{T-106}{3,42}}\right),\;70\;{}^{\circ}C\leq T<100\;{}^{\circ}C\\ 7.053-0.064096\cdot T,\;100\;{}^{\circ}C\leq T<104\;{}^{\circ}C\\ 0.778\cdot e^{-\frac{T-80}{34.37}},\;T\geq 104\;{}^{\circ}C \end{array}\right. \end{array}$$

**Figure 10:**
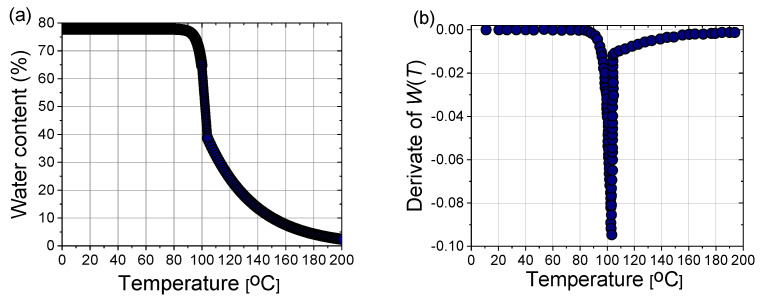
(**a**) Variation of the water content with the temperature *W*(*T*) according to (17) and (**b**) the first derivative of *W*(*T*) necessary for the calculation of the effective specific heat (18).

In a steady state, the liver tissue contains approximately 78% water by mass (Figure 10a). Therefore, its thermal properties are similar to those of water [40]. As a result of evaporation at temperatures above 100 °C, the water content of the tissue may decrease to less than 20% by mass, leading to drastic changes in the dielectric properties of the tissue and the significant penetration of microwaves. The effect of water evaporation can be included in the bio-heat Equation (8) by replacing specific heat *c* with an effective value *c’*:

(18) $$c^{\prime }=c-\frac{\alpha }{\rho }\frac{\partial W}{\partial T},$$

where the derivative of *W*(*T*) is plotted in Figure 10b, and *α* = 2260 (kJ/kg) is the latent heat constant [40]. 

Damage to biological tissues depends on both temperature and time. The most well-known expression for determining tissue injury *Ω* is the Arrhenius form [107]:

(19) $$\Omega \left(t\right)=\int_{0}^{t}A\exp\left(-\frac{\Delta E}{RT}\right)dt,$$

with the frequency factor *A,* temperature *T*, and activation energy for irreversible damage reaction Δ*E*. *R* is the gas constant. 

## 4. Modeling of Microwave Ablation in the Treatment of Hepatocellular Carcinoma

Hepatocellular carcinoma (HCC) is the most common form of liver cancer, constituting > 90% of primary liver malignancy [108,109,110]. In terms of HCC mortality, the 3−year survival rate is only 12.7% [111,112]. Although HCC is the fourth most common cause of cancer-related deaths worldwide, therapeutic options remain insufficient. One of the most promising therapies for HCC is MWA at 2.45 GHz as a rapid treatment with a short recovery [9,113]. The development of mathematical models enables the determination of the optimal conditions necessary for planning patient-specific MWA procedures to be as effective as possible for treating HCC [50,53]. 

**Figure 11:**
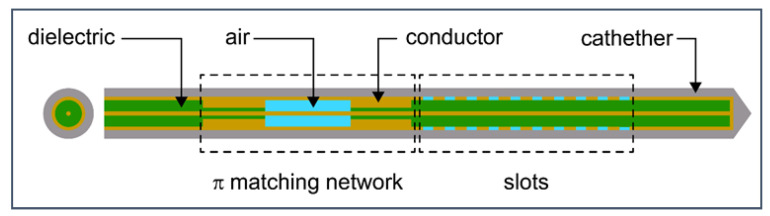
Schematic of 10-slot microwave antenna. The width of a single slot is 0.6 mm, whereas the distance between two slots is 0.8 mm. Finely tuned impedance matching preserves the surrounding tissues.

The 3D models of the MW applicator (antenna) and the tumor without homogeneity assumptions are the most realistic representations of the physical problem. However, the complexity and computational resource consumption limit the number of 3D models. Hence, most numerical studies are 2D models rotated around an axis to emulate 3D models that are often far from reality. To overcome the limitations and deficiencies of the 2D models, a full 3D model was developed by considering all the details described in the previous sections. The 3D finite element method (FEM) within COMSOL Multiphysics [114] was applied to solve the system of coupled equations corresponding to the electromagnetic field and heat transfer. In the model, Pennes’ bio-heat equation is used, taking into account the temperature dependence of thermal conductivity and effective specific heat. The temperature dependence of electrical conductivity and permittivity of healthy and tumoral tissues is included in the calculation of the electromagnetic field. A compact 10-slot antenna with several periodic elements (Figure 11) was immersed in tissue [50,53]. 

To make the model as realistic as possible, we did not use a spherical tumor geometry, as was typically performed. Instead, we used realistic tumor geometry based on data from the 3D-IRCADb-01 database, which comprises several sets of patients’ CT scans manually segmented by clinical experts [115]. The simulation parameters characteristics for healthy liver, malignancy, and blood are listed in Table 1 [50,53,102].

### 4.1. Results

#### 4.1.1. Hepatocellular Carcinoma of Patient 16 in the 3D-IRCADb-01 Database

**Figure 12:**
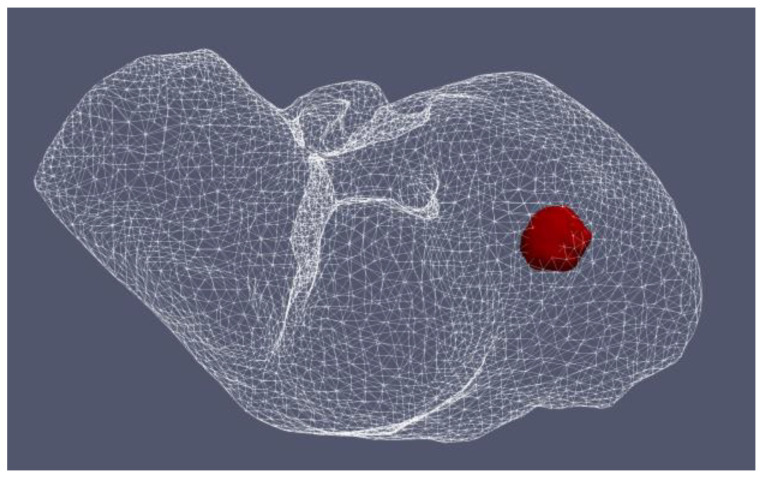
Three-dimensional simulation model of HCC (solid red surface), which belonged to patient 16 in the 3D-IRCADb-01 database [115], and its position in the liver (triangulated surface).

**Figure 13:**
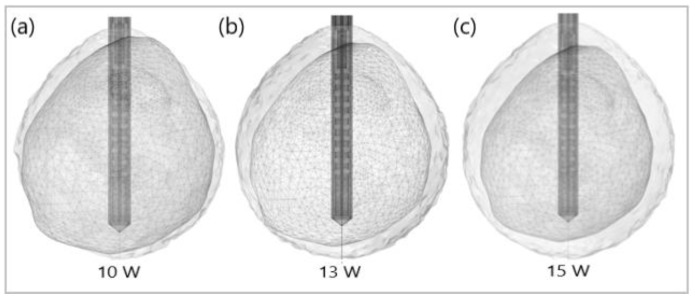
Isocontours that are related to ablated regions (solid gray surfaces) around the HCC (triangulated surface) after 600 s of microwave ablation at 2.45 GHz with an input power of (**a**) 10 W, (**b**) 13 W, and (**c**) 15 W.

Figure 12 shows the liver (triangular surface) with early-stage HCC (1.64 cm × 1.71 cm × 3.81 cm) (solid red surface) of patient 16 (male, born in 1950) in the 3D-IRCADb-01 database [115]. The optimal value of the input power is the value required for treating the entire tumor with minimal damage to the healthy surrounding tissue. In the case of HCC in patient 16 [115], the optimal input power can be evaluated from the isocontours associated with the fraction of damage equal to 1, as shown in Figure 13. When a power of 10 W was applied, the tumor was not completely ablated. At 15 W, the ablation zone encompasses not only the whole tumor but also significant damage to healthy tissue. The application of 13 W provides treatment for the entire tumor with minimal damage to the surrounding tissue [50]. Because the shape and size of the tumor significantly affect the proper choice of input power, it should be estimated before the procedure begins to attain the desired ablation zone.

**Figure 14:**
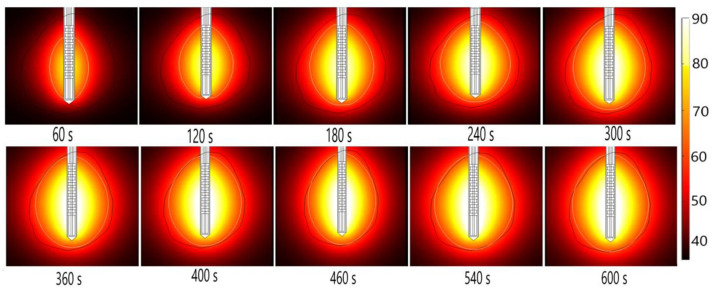
The temperature distribution during microwave ablation (in °C) when an early-stage HCC [115] is exposed to a frequency of 2.45 GHz and an input power of 13 W. The boundary of the tumor tissue is represented by a black line.

Energy radiated by the MW antenna is absorbed and converted into thermal energy. The temporal evolution of the temperature distribution during MWA is shown in Figure 14. Black and white lines mark the boundary of the tumor and the lethal isotherm at 60 °C, respectively. The 60 °C isothermal contours are correlated to the lesion size and shape of the ablated tissue [50]. Blood perfusion limits the extent of the heating. 

**Figure 15:**
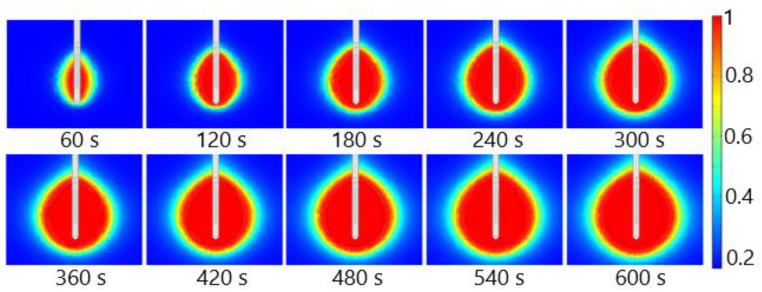
The fraction of tissue damage during microwave ablation of HCC [115] at a frequency of 2.45 GHz and an input power of 13 W. The black line denotes the boundary of the tumor.

The fraction of necrotic tissue during MWA at 2.45 GHz and a power of 13 W is shown in Figure 15. A multislot antenna produces near-spherical ablation zones with damage concentrated around the tip and slots of the antenna [50]. The 10-slot antenna produces localized near-spherical heating distributions. Two distinct heating zones can be distinguished [8]. The active heating zone is located inside the tumor close to the antenna where the intensity of energy is high and its absorption by tissue is fast. The passive zone is far from the antenna in the region with low energy intensity.

**Figure 16:**
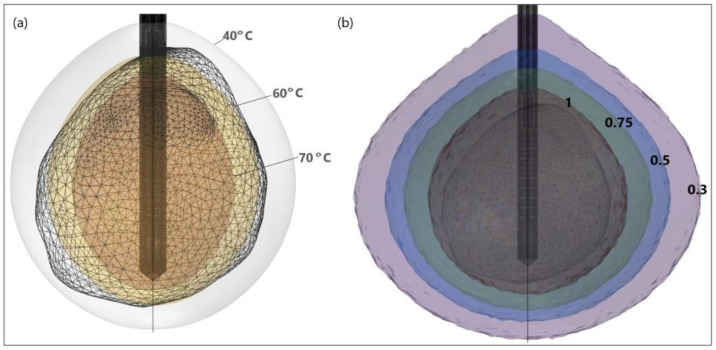
HCC (triangulated surface) [115] and isocontours that correspond to (**a**) temperatures of 40 °C, 60 °C, and 70 °C, and (**b**) fractions of damage of 0.3, 0.5, 0.75, and 1.

It is evident from the isocontours related to different temperatures (Figure 16a) that, when approaching the antenna, the heat source is stronger, and the temperature is higher. Isocontours associated with different fractions of damage (Figure 16b) illustrate that complete ablation of the tumor was achieved for a fraction of damage of 1 [50].

#### 4.1.2. Hepatocellular Carcinomas of Patient 1 in the 3D-IRCADb-01 Database

**Figure 17:**
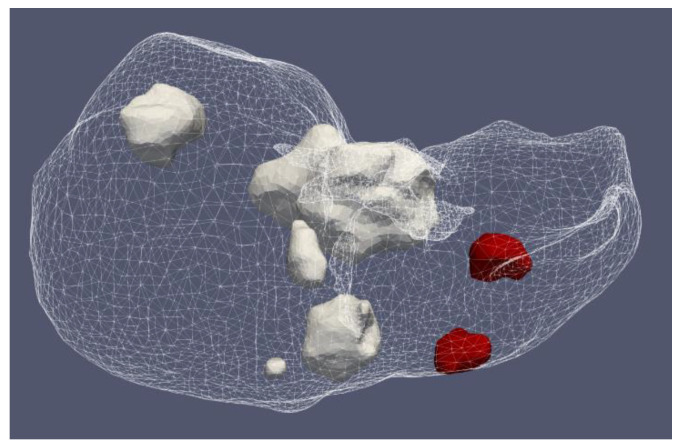
Positions of seven HCCs (solid surfaces) in the liver (triangulated surface) of patient 1 in the 3D-IRCADb-01 database [115].

**Figure 18:**
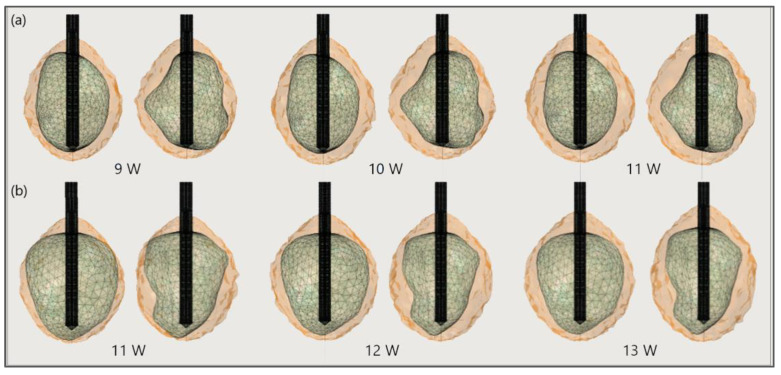
The ablated regions (solid light brown surface) around the front (left) and back (right) sides of tumors (triangulated surface) (**a**) 1.07 [115] and (**b**) 1.03 [115] after 600 s of microwave ablation at 2.45 GHz and different values of the input power.

Figure 17 shows the liver and severe HCCs of patient 1 (female born in 1940) in the 3D-IRCADb-01 database [115]. Simulations were carried out for two of them (solid red surfaces) labeled as 1.07 (1.74 cm × 1.53 cm × 2.10 cm) and 1.03 (1.78 cm × 1.97 cm × 2.27 cm). First, the optimal values of the input power were determined for both HCCs. Because of the irregular shapes of the tumors, both sides—the front (left) and back (right)—of the tumors are shown. For tumor 1.07 [115] (Figure 18a), an input power of 9 W did not ensure complete ablation of the tumor (backside). When 11 W was applied, the ablation zone enclosed the entire tumor, and a large amount of healthy tissue was damaged. The isocontour that ensures necrosis of the entire tumor preserving healthy tissue was achieved for an input power of 10 W [53]. By using a similar procedure, it was found that the optimal input power for HCC 1.03 [115] is 12 W (Figure 18b).

**Figure 19:**
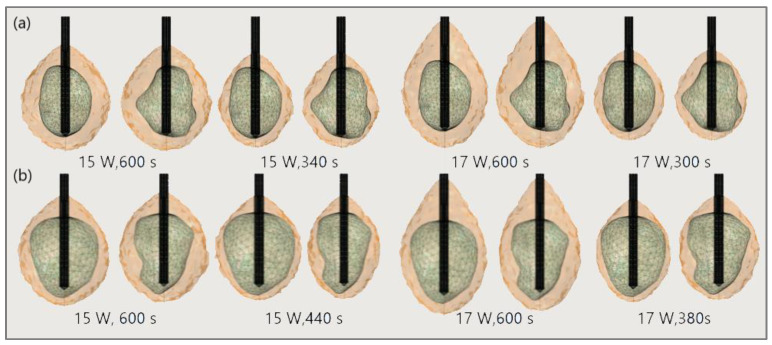
Isocounturs representing the ablated regions (solid light brown surface) around the front (left) and back (right) sides of tumors (triangulated surface) (**a**) 1.07 [115] and (**b**) 1.03 [115] for input powers of 15 W and 17 W and various duration of microwave ablation.

**Figure 20:**
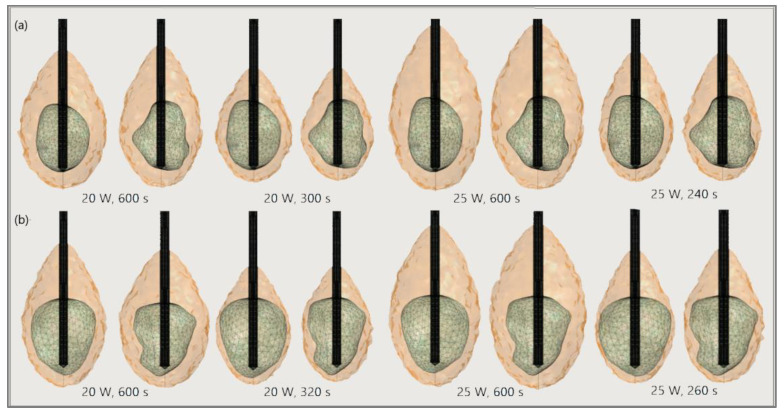
The ablated regions (solid light brown surface) around the front (left) and back (right) sides of tumors (triangulated surface) (**a**) 1.07 [115] and (**b**) 1.03 [115] for input powers of 20 W and 25 W and various ablation times.

The significance of the proper choice of optimal values of the input power and ablation time is demonstrated in Figure 19. The ablation time decreased with an increase in the input power. Damage to healthy tissue around tumors applying a power of 15 W or 17 W during a shorter ablation time is similar to those obtained for 10 W (for tumor 1.07 [115]) and 12 W (for tumor 1.07 [115]) after 600 s [53]. However, higher input power and shorter ablation time are not always related to the safest MWA treatment. Further increases in the input power cause significant damage to healthy tissue despite the shorter ablation time, as shown in Figure 20. For higher input power, ablation zones became more elongated with a greater length along the shaft of the antenna causing unavoidable damage to the healthy tissue [53].

**Figure 21:**
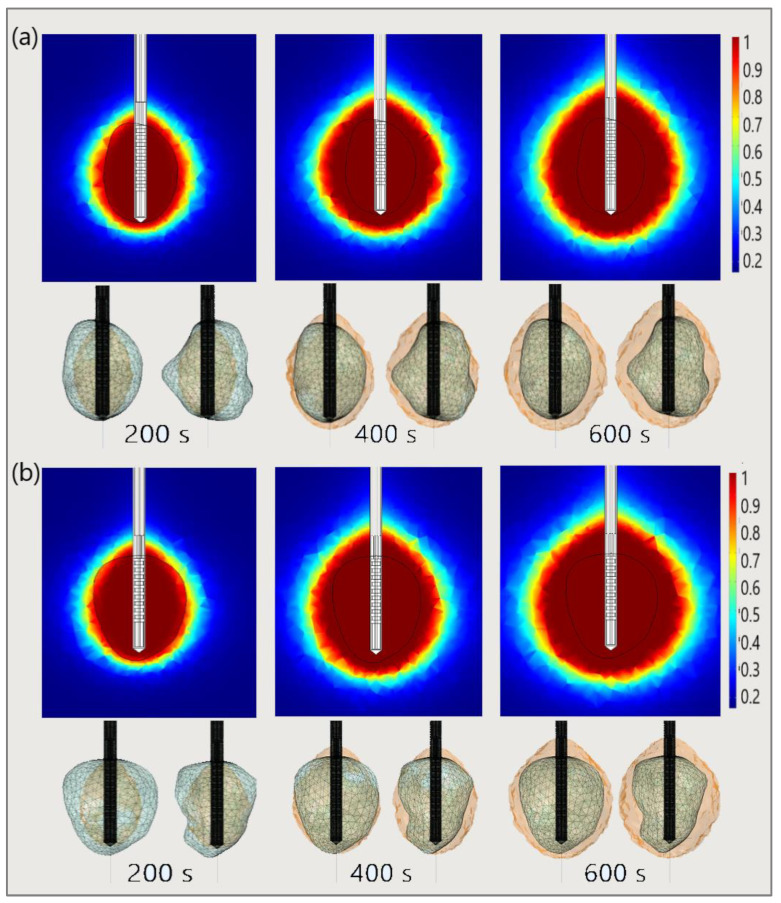
The time evolution of the necrotic tissue tumors (**a**) 1.07 [115] and (**b**) 1.03 [115], after ablation times of 200 s, 400 s, and 600 s. Necrotic tissue in the cut plane (x = 0) (upper figures) and the front and back sides of the tumors (triangulated surfaces) (lower figures).

Finally, to demonstrate the necessity of performing 3D simulations, Figure 21 shows the necrotic tissue and time required for the complete ablation of tumors (a) 1.07 [115] and (b) 1.03 [115]. The ablation zones plotted in the upper figures (in the cut plane x = 0) may not precisely represent tissue necrosis. The upper figures indicate that the entire tumor was ablated after 400 s. However, both sides of the tumor (lower figures) suggest that they were completely ablated after 600 s [53].

## 5. Discussion

Since the thermal spread in biological tissue is difficult to measure, the development of predictive models is essential. For studying heat transport in complex heterogeneous systems such as biological tissues, macroscale models must be used. The macroscopic point of view is important foremost to overcome possible complications due to a large number of particles at the molecular scale or the compound microscale structure of tissues. Proper separation of the length scales represents a crucial step in establishing a macroscale representation that is identical to the microscale behavior. Beyond macroscopic models, some researchers have attempted to provide a link between microscopic processes and the overall dynamics of tissues. Based on the mixture theory and poroelasticity concepts, tissues are represented as a mixture of interacting solid and fluid phases, which enable investigation of the biological tissues at a fundamental level. Micromechanical effects at the cellular level also must be included for determining the heat transport and thermal damages for which satisfactory models still do not exist. However, researchers must have the disposal of novel computer approaches directed toward the development of more sophisticated tissue models.

Computer modeling is proven to be an effective tool to improve the performance of MWA. Most of the existing numerical models of MWA are 2D axis-symmetric based on the assumption of a homogeneous medium reducing the problem from 3D to 2D. Taking into account that each tumor has a different shape and size, the development of 3D predictive models of the MWA procedure including all details of the targeted tissue characteristics and the antenna design is a prerequisite for further ablation studies with a promising possibility of application in the treatment planning adjusted for each patient.

## 6. Conclusions and Perspectives

Microwave ablation therapy has become an important topic in medicine, and numerous investigations on the applications of heat transfer to biological tissues have been conducted in the last few decades. Numerical methods may significantly influence patient care by creating predictive models from procedural planning to execution. Furthermore, the lack of experimental results in this field emphasizes the need for mathematical models. Understanding the science underlying ablation therapy is crucial for developing accurate models of tissue and heat processes. For microwave ablation simulations, every mathematical model must contain three fundamental components that depend on the tissue characteristics of an individual patient. Currently, complex heat transfer models rely on the porosity concept, leading to two “bioheat” equations for the tissue and blood temperatures.

In this article, we review the current status of numerical methods for modeling microwave ablation procedures. We reviewed different models and theories proposed to describe tissue and heat transport. We compare them to highlight their differences and identify important trends. We have also summarized numerous microwave antenna designs developed over the last few decades including a recent multi-slot antenna that offers the best performance and provides near-spherical ablation zone patterns. To illustrate the application of the reviewed numerical methods, we present the results of three-dimensional simulations of real Hepatocellular Carcinomas from the 3D-IRCADb-01 database. The primary aim was to create a realistic simulation model that could predict ablation results. The final goal is to transform the treatment plan based on the simulation outputs into a minimally invasive and safe microwave ablation procedure. Therefore, several questions and challenges remain. The main reason for this is whether and how antenna design alters the dielectric and other properties of the tissue during microwave ablation. Finally, this work focused on microwave ablation, but with numerous other operating modes, these concepts and theories need to be expanded. All these challenges may lead to exponential progress in the computational modeling of microwave tissue ablation.

## Figures and Tables

**Table 1:** Parameters of biological materials (healthy liver tissue, liver tumor lesions, and blood) used in the simulations [50,53,102].

Parameter	Value
Healthy liver tissue	
Density	1079 kg/m^3^
Thermal conductivity	0.52 W/m °C
Specific heat	3540 J/kg °C
Tumoral liver tissue	
Density	1040 kg/m^3^
Thermal conductivity	0.57 W/m °C
Specific heat	3960 J/kg °C
Blood	
Density	1060 kg/m^3^
Thermal conductivity	0.5 W/m °C
Specific heat	3600 J/kg °C
Temperature	37 °C

## Data Availability

Data are contained within the article.
